# Prenatal Magnesium Sulfate Exposure Is Not Associated with Different Neurodevelopmental Outcomes by Sex in Extremely Preterm Infants

**DOI:** 10.3390/brainsci15121273

**Published:** 2025-11-27

**Authors:** Kate F. DiNucci, Tessa C. Rue, Olivia C. Brandon, Kylie A. Corry, Dennis E. Mayock, Patrick J. Heagerty, Sandra E. Juul, Thomas R. Wood

**Affiliations:** 1 Division of Neonatology, University of Washington, Seattle, WA 98105, USAsjuul@uw.edu (S.E.J.); 2 Department of Biostatistics, University of Washington, Seattle, WA 98105, USA; 3 Institute on Human Development and Disability, University of Washington, Seattle, WA 98105, USA

**Keywords:** magnesium sulfate, neonatal, neuroprotection, neurodevelopment, preterm

## Abstract

**Background/Objectives**: Magnesium sulfate (MgSO_4_) has historically been used in obstetrics as a tocolytic and to prevent eclamptic seizures. MgSO_4_ has also been investigated as a potential neonatal neuroprotectant for infants born preterm. However, randomized controlled trials of prenatal MgSO_4_ have shown mixed results, with single-center observational studies also suggesting differential effects by sex. We sought to evaluate sex-dependent associations between prenatal MgSO_4_ exposure and standardized neurodevelopmental outcomes in a large, multi-center cohort of extremely preterm neonates (24–0/7 to 27–6/7 weeks’ gestation) from the Preterm Erythropoietin Neuroprotection Trial (PENUT). **Methods**: The relationship between maternal MgSO_4_ exposure and neurodevelopmental outcomes assessed at 2 years using the Bayley Scales of Infant and Toddler Development Index, 3rd edition was examined by sex in *n* = 666 infants (*n* = 328 female, *n* = 338 male). To account for confounding by indication, we performed both matching and inverse probability weighting using 17 maternal predictors of MgSO_4_ exposure. **Results**: In both unadjusted and adjusted (weighted and matched) analyses, no relationship between MgSO_4_ exposure and neurodevelopmental outcomes was seen, either overall or by sex. **Conclusions**: This study reaffirms the safety of MgSO_4_, but appropriate clinical trials of MgSO_4_ in extremely preterm infants are still required to better understand any effects on neurodevelopmental outcomes.

## 1. Introduction

Magnesium sulfate (MgSO_4_) has a long history in obstetrics for the prevention of eclamptic seizures and as a tocolytic to slow the onset of preterm labor [1,2,3]. As a non-competitive inhibitor of the *N*-methyl-D-aspartate (NMDA) glutamate receptor, MgSO_4_ regulates intracellular calcium in the brain, increasing the seizure threshold [3]. Similarly, MgSO_4_’s tocolytic effects are linked to its ability to decrease intracellular calcium levels in uterine muscles in the setting of preterm labor.

In the presence of acute brain injuries such as neonatal hypoxia–ischemia (HI), over-activation of NMDA receptors leads to excess intracellular calcium and a cascade of mitochondrial dysfunction, free radical production, inflammation, and cell death [4]. As a modulator of NMDA activity, MgSO_4_ has therefore been investigated as a potential neonatal neuroprotectant. In a rabbit model of spinal cord ischemia, MgSO_4_ in combination with therapeutic hypothermia administered before the insult was found to decrease the degree of irreversible damage [5]. MgSO_4_ has also been shown to protect against hypoxia-induced injury in rat hippocampal slices [5] and reduce the effect of NMDA-mediated excitotoxicity in a rat model of perinatal brain injury [6]. However, a review of early preclinical studies suggested that many studies employing MgSO_4_ failed to control for hypothermia, which is known to be neuroprotective [7], while studies that did control for temperature reported a lack of neuroprotection [8]. Furthermore, in a preterm fetal sheep model of asphyxia, MgSO_4_ administration failed to protect against hypoxic injury and intensified oligodendrocyte loss [9].

MgSO_4_’s prominence in obstetrics has also facilitated observational studies on prenatal administration of MgSO_4_ in infants born preterm. A recent meta-analysis of 11 observational studies found an association between MgSO_4_ exposure and lower mortality (risk ratio [RR], 0.73; 95% confidence interval [CI] 0.61–0.89) as well as a decreased odds of cerebral palsy (CP) (odds ratio [OR], 0.64; 95% CI 0.47–0.89) in preterm infants [10].

Throughout the 1990s and 2000s, researchers conducted five major randomized controlled trials to assess MgSO_4_ as a preterm neonatal neuroprotectant [11,12,13,14]. Multiple meta-analyses of these trials consistently found that MgSO4 had no significant effect on mortality or neonatal morbidities such as necrotizing enterocolitis, intracranial hemorrhage, or chronic lung disease. However, nearly all the meta-analyses found a significant risk reduction for CP [11,12,13,14].

The efficacy and applicability of MgSO_4_ continues to be a topic of interest in neonatal neuroprotection research. For example, a retrospective unadjusted single-center analysis exploring the relationship between prenatal MgSO_4_ administration and cognitive and language outcomes at 18 months corrected gestational age in preterm neonates suggested there was a sex-dependent effect of MgSO_4_ associated with improved neurodevelopmental outcomes in females, but worse outcomes in males [15]. As the importance of possible sexual dimorphism in neonatal outcomes and treatment responses is increasingly being recognized, the objective of the current study was to evaluate the sex-dependent effects of fetal MgSO_4_ exposure, including confounding by indication, on standardized neurodevelopmental outcomes in a large, multi-center cohort of extremely preterm (EP) neonates from the Preterm Erythropoietin Neuroprotection Trial (PENUT) [16].

## 2. Materials and Methods

This study follows the Strengthening the Reporting of Observational Studies in Epidemiology (STROBE) reporting guideline for observational studies [17]. PENUT was a phase 3, placebo-controlled, randomized clinical trial of erythropoietin in EP newborns in 19 academic centers and 30 neonatal intensive care units (NICUs) in the US [16]. PENUT included infants born 24-0/7 to 27-6/7 weeks’ gestation and enrolled within 24 h of birth. Exclusion criteria for PENUT included known life-threatening anomalies, chromosomal anomalies, disseminated intravascular coagulopathy, twin-to-twin transfusion, polycythemia, hydrops fetalis, or known congenital infection, as previously described [16]. Written informed consent for participation in the PENUT Trial was obtained from the infant’s parents or legal guardian. The PENUT Trial was approved by the University Washington Institutional Review Board and the institutional review boards of each recruiting site. Detailed demographic and clinical data were collected during the initial hospital stay. Maternal age, ethnicity, race, education, and infant information was collected via questionnaires at enrollment.

In PENUT, neurodevelopmental outcomes were assessed at 20 to 33 months postmenstrual age using Bayley Scales of Infant and Toddler Development Index 3rd edition (BSID-III) scores for motor, cognitive, and language skills. Gross Motor Function Classification System (GMFCS) levels were also assessed, which are commonly used for prognostication of CP in children [18]. All infants from PENUT who had documentation of prenatal MgSO_4_ exposure status and survived to receive assessment with at least one BSID-III subscale were eligible for inclusion in this study.

All statistical analyses were conducted using R (R Foundation for Statistical Computing, Vienna, Austria), version 4.4.3. First, we generated a propensity score for MgSO_4_ exposure at the maternal level using a logistic model with the following covariates: gestational age at birth, maternal age, ethnicity, race, education, obesity, gestational diabetes, hypertension, prenatal care, multiple birth, preterm labor, premature rupture of membranes (PROM), prenatal antibiotics, prenatal steroids, C-section, and small for gestational age (SGA) status (weight below the 10th percentile for gestational age) [19].

To estimate the effects of MgSO_4_ exposure on BSID scores, we used Generalized Estimating Equations (GEE) with an independent correlation structure. We obtained robust standard errors to account for correlation among multiples within a birth. The mean model also included infant sex and allowed for different MgSO_4_ exposure effects by sex. We used two different approaches to control for potential confounding by indication: weighting and matching.

In the weighted analysis approach, we used data from all infants but weighted their contribution inversely to their propensity to be exposed or unexposed to MgSO_4_ in utero. This approach yields an estimate of the average effect of exposure among all infants. In the matched approach, we performed 2:1 matching of exposed to unexposed mothers using the propensity score, with no replacement. We also included the propensity score as a covariate in the GEE mean model. The estimated MgSO_4_ effect obtained from the matched sample is the average effect of exposure among unexposed infants.

**Figure 1:**
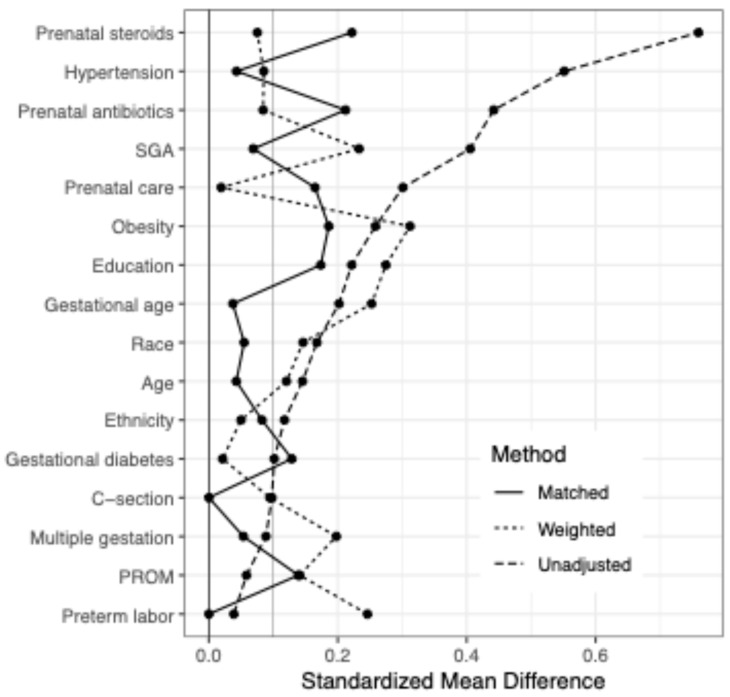
Difference between exposed and unexposed MgSO_4_ groups across maternal/birth covariates when entire sample weighted using Inverse Probability Weighting (Weighted) vs. not weighted (Unadjusted) vs. restricted to matched sample (Matched).

To compare the success of the two approaches at removing potential confounding, we plotted the standardized mean difference between exposed and unexposed groups for each covariate for (a) the entire unadjusted sample, (b) the entire sample weighted using Inverse Probability Weighting on the propensity score, and (c) the sample matched on the propensity score (Figure 1). The standardized mean difference acts as an effect measure of the covariate between exposure groups, with normalization by standard deviation. A probability (*p*) level of less than 0.05 was considered significant. Following adjustment, the standardized mean difference between groups by covariates was largely reduced, particularly regarding some of the largest unadjusted differences including prenatal steroids, hypertension, antibiotics and SGA diagnosis.

## 3. Results

A total of 666 infants from 595 mothers were included. Of the total sample, 562 were exposed to MgSO_4_ (84.38%). Gestational age ranged from 24 to 27 completed weeks, and the mean birthweight was 813 g (187.9).

### 3.1. Relationship Between Maternal Characteristics and Prenatal MgSO_4_ Exposure

Baseline maternal characteristics, separated by all infants and those included in matched analyses, are outlined in Table 1. In the unmatched group, there was no association between MgSO_4_ exposure and maternal age at birth, ethnicity, race, or education level (Table 1). Further, there was no association between MgSO_4_ administration and gestational diabetes, multiple gestation, preterm labor, PROM, C-section, or gestational age at birth. In the unmatched MgSO_4_ exposure group, there was significantly greater incidence of prenatal SGA diagnosis (*p* = 0.01), prenatal steroid exposure (*p* < 0.001), prenatal antibiotics (*p* = 0.001), maternal hypertension (*p* < 0.001), maternal obesity (*p* = 0.041) and prenatal care (*p* = 0.002) compared to the non-MgSO_4_ group (Table 1A).

### 3.2. In Unadjusted Analyses, MgSO_4_ Is Not Associated with Neurodevelopmental Outcomes in Either Sex

**Figure 2:**
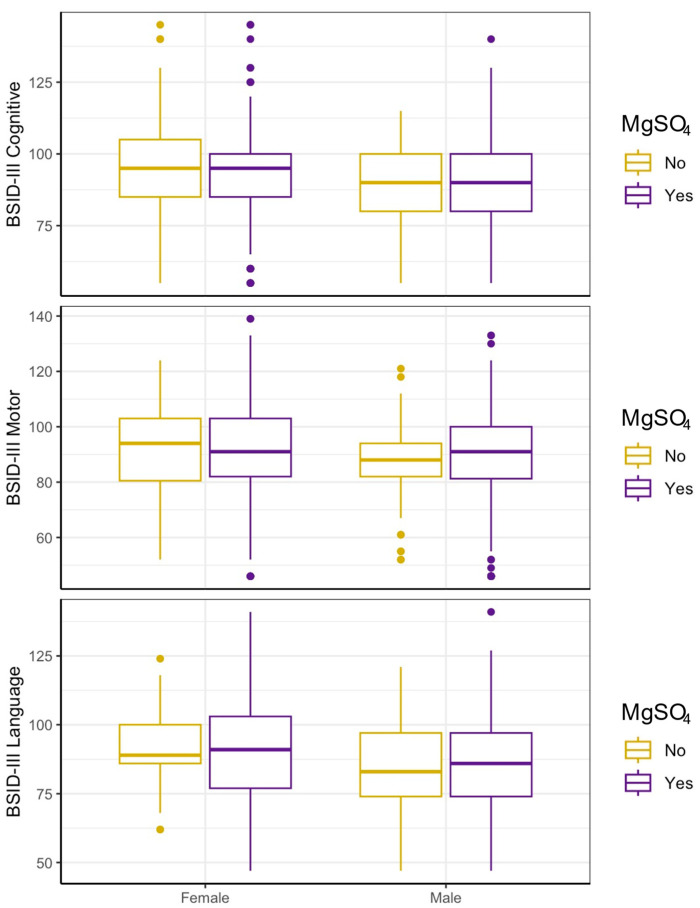
MgSO_4_ exposure and BSID outcomes by sex–unadjusted. Infant outcomes by sex and MgSO_4_ exposure (*n* = 660). Motor score missing *n* = 10; Language score missing *n* = 13. No significant difference between outcomes were seen by MgSO_4_ exposure, including when stratified by sex.

Initial analyses were conducted in an unadjusted manner to assess the baseline relationship between antenatal MgSO_4_ exposure and BSID-III scores (cognitive, motor, and language). No differences between mean BSID-III cognitive, motor, or language scores for the MgSO_4_ exposure versus non-MgSO_4_ exposure groups were seen. We further stratified each group by sex to assess sex-dependent associations of antenatal MgSO_4_ exposure. Expressed as the standardized mean difference (d) in points on the BSID-III subscales, there was no effect of antenatal MgSO_4_ exposure for females across BSID-III cognitive (−1.67, 95% CI −6.9–3.57), motor (d −0.18, 95% CI −4.79–4.42), and language (d −0.15, 95% CI −4.37–4.07) scores. Similarly, males displayed no significant estimated effect of MgSO_4_ exposure on cognitive (d −0.83, 95% CI −6.52–4.86), motor (d 0.95, 95% CI −4.79–6.69), or language score (d 1.38, CI −4.86–7.63). Accounting for interaction by sex yielded consistent, non-significant results for all BSID-III subscales (cognitive: d 0.84, 95% CI −6.84–8.53, motor: d 1.13, 95% CI −6.17–8.43, language: d 1.53, 95% CI −5.94–8.99) (Table 2). For all categories of BSID-III scores, there was no significant difference by sex for antenatal MgSO_4_ exposure (Figure 2). GMFCS scores were used as a predictor of CP for both groups. In both males and females, there was no significant relationship between prenatal MgSO_4_ administration and GMFCS scores (Table 3).

### 3.3. MgSO_4_ Is Not Associated with Neurodevelopment in Either Sex After Accounting for Indication

For additional statistical sensitivity, a propensity score was generated to create an inverse probability weighted statical analysis of BSID-III scores versus MgSO_4_ exposure (*n* = 666) based on the associations between maternal characteristics and prenatal MgSO_4_ exposure. Additionally, a 2:1 matched analysis was conducted based on exposed (*n* = 208) to unexposed (*n* = 104) mothers. The effectiveness of adjusted methods at reducing confounding by indication was assessed by plotting standardized mean differences between exposure groups for each covariate (Figure 1). After both weighting and matching, group imbalances were improved, suggesting that the majority of confounding by indication was accounted for using these methods. Similarly, there were no significant differences in maternal characteristics between the MgSO_4_-exposed and -unexposed groups after matching (Table 1B).

When using weighting, effect estimates of MgSO_4_ exposure for females yielded no significant difference for any subscales of BSID-III (cognitive: d 0.57, 95% CI −8.92–10.06, motor: d 3.51, 95% CI −3.62–10.64, language: d 0.4, 95% CI −4.74–5.53). Males also displayed no significant effect of MgSO_4_ administration for BSID-III scores in all categories (cognitive: d −1.72, 95% CI −7.63–4.19, motor: d −0.05, 95% CI –6.16–6.06, language: d 0.34, 95% CI −4.74–5.53). Additionally, no significant interaction for the effect of MgSO_4_ on outcomes by sex was seen (cognitive: d −2.29, 95% CI −13.44–8.87, motor: d −3.56, 95% CI −12.91–5.79, language: d −0.05, 95% CI −9.03–8.92) (Table 2).

**Figure 3:**
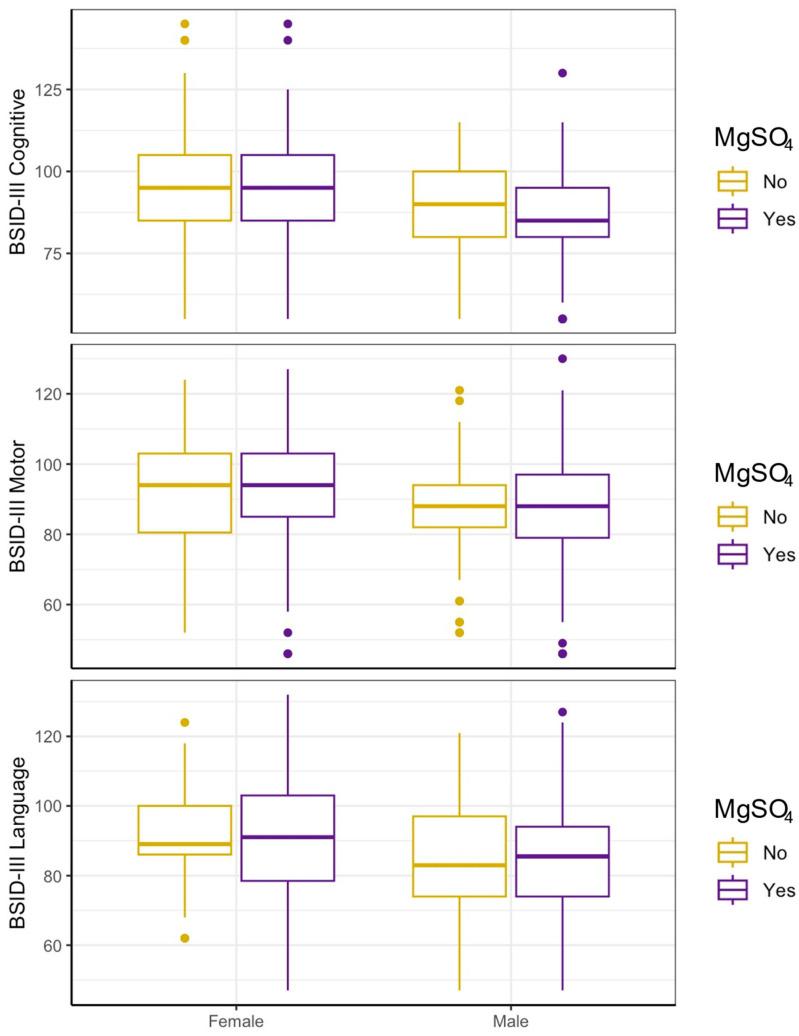
MgSO_4_ exposure and BSID outcomes by sex–matched. Infant outcomes by MgSO_4_ exposure and baby sex in a sample matched on all maternal/birth covariates except for chorioamnionitis (*n* = 312, 2:1 exposed/unexposed). No significant difference between outcomes by MgSO_4_ exposure was seen, including when stratified by sex.

Similarly, in the 2:1 matched analysis, MgSO_4_ exposure was not associated with any differences in BSID-III scores in females (cognitive: d −0.91, 95% CI −7.22–5.41, motor: d 1.73, 95% CI −63.66–7.13, language: d −0.36, 95% CI −5.71–5) or males (cognitive: d −2.53, 95% CI −8.59–3.54, motor: d −0.38, 95% CI −6.48–5.72, language: d 0.15, 95% CI −6.53–6.84). There was also no significant effect of MgSO_4_ when further accounting for interaction by sex (cognitive: d −1.62, 95% CI −10.1–6.86, motor: d −2.12, 95% CI −10.08–5.84, language: d 0.51, 95% CI −7.72–8.74) (Table 2, Figure 3). Similarly, GMFCS scores displayed no association with MgSO_4_ exposure in both females (*p* = 0.54) and males (*p* = 0.74) (Table 3).

## 4. Discussion

In this study, we examined the association between antenatal MgSO_4_ exposure, neurodevelopmental outcomes, and sex from a large, multi-center study population of EP infants. To minimize confounding by indication, we performed inverse probability weighting and 2:1 (exposed/unexposed) matching. We analyzed 17 maternal/birth covariates, 11 of which had a standardized mean difference greater than 0.1 between exposure groups in the unadjusted analysis, suggesting that confounding by indication may have contributed to previous findings on the effects of MgSO_4_ on infant outcomes. Across all analysis methods, including after accounting for confounding, the results were concordant with an absence of a sex-dependent association between antenatal MgSO_4_ administration and neurodevelopment.

MgSO_4_ has been recommended by the International Federation of Gynecology and Obstetrics for use as a preterm neuroprotectant in pregnancies <30 weeks GA at risk of imminent preterm birth, with consideration warranted for pregnancies <32–34 weeks GA, administered intravenously within the 24-h window prior to birth [20]. Recommendations for use are supported by consistent, significant reduction in risk of CP, without a reduced risk of mortality or non-CP morbidities [11,12,13,14]. The mechanisms of neuroprotection by MgSO_4_ are hypothesized to be due to its role as a non-competitive NMDA receptor antagonist, thereby modulating extracellular glutamate and calcium influx—species linked to cytotoxic and apoptotic pathways in preterm brain injury [1,4,21,22,23]. The incidence and severity of brain injury increase with decreasing GA at birth [24]. Perinatal inflammation and HI are commonly associated with such injuries [25,26]. During the primary energy failure phase of HI, the activation of NMDA of receptors leads to a marked increase in intracellular Ca^2+^ [27]. Characterized by acute energy depletion, the initial insult compromises mitochondrial function and promotes the production of harmful free radicals, resulting in apoptotic and necrotic neuronal cell-death. By reducing the probability of voltage-gated Ca^2+^ ion channels opening, MgSO_4_ effectively limits the injury cascade early in the process of HI [4]. In the hours and days following the initial injury, a secondary injury phase can emerge due to reperfusion [28,29]. Cells are overwhelmed and unable to safely utilize the amount of reintroduced oxygen, leading to the production of harmful reactive oxygen species (ROS). ROS stimulate lipid peroxidation, DNA damage, and the production of pro-inflammatory agents. Some studies indicate potential protective effects of MgSO_4_ against the inflammatory damage of secondary energy failure by reducing the production of cytokines such as interleukin-6 (IL-6) and tumor necrosis factor (TNF) [4,30,31,32,33,34]. However, prenatal MgSO_4_ is unlikely to influence the tertiary phase of injury characterized by prolonged inflammation and impaired neurogenesis, particularly due to microglia overactivation [35,36,37,38,39,40]. The resultant white matter abnormalities from oligodendrocyte damage following HI have been shown to be predictive of neurodevelopmental outcomes at 2, 4, and 6 years adjusted age [41,42,43]. However, neuroimaging at term-equivalent age following prenatal MgSO_4_ administration in a moderately and very preterm cohort revealed that MgSO_4_ did not promote myelination in pathways related to motor and cognitive function [44].

It has been well-established that preterm birth and perinatal HI are risk factors for CP, though the precise cellular pathophysiology remains complex [26]. Investigation of potential predictive biomarkers of CP has suggested an association between cytokines, mitochondrial regulation, and inflammatory response pathways [45,46,47,48]. A study in EP infants suggested that repeatedly elevated levels of inflammation-related proteins including IL-6 and TNF led to an increased risk of CP diagnosis at 2 years of age [49]. Though the etiology of CP is likely multifactorial, similarities between identified biomarkers and neuromodulatory mechanisms of MgSO_4_ in the HI primary and secondary injury phases may help to explain the previously established findings of reduced odds of CP following antenatal MgSO_4_ exposure. In contrast, the reported lack of effect of MgSO_4_ on non-CP morbidities and neurodevelopmental outcomes may be indicative of unmitigated tertiary injury, especially with respect to white matter maturation.

We did not observe a significant association between GMFCS scores and MgSO_4_ exposure, which is inconsistent with previously reported risk reductions in CP from preterm observational and clinical studies of prenatal MgSO_4_ administration [10,11,12,13,14]. We hypothesize this disparity may be due to differences in study populations. The aforementioned study populations primarily investigated MgSO_4_ administration in moderate and very preterm infants, and in some cases, reported no significant decrease in CP when conducting sub-group analyses of GA < 30 weeks at trial entry [12]. The lack of representation of EP infants is unsurprising given that these studies were conducted throughout the 1990s and 2000s, when mortality and morbidity rates for EP infants were over 65%, which has since decreased significantly [50]. EP infants remain particularly vulnerable, and most will spend the entirety of the third trimester developmental period in the NICU with support required for immature lungs, fragile vasculature, and nutritional maintenance [51]. Lymphopenia and immune dysregulation associated with EP increase susceptibility to infection and inflammation levels [52]. Compared to moderate preterm infants, EP infants have significantly higher mortality and sequalae of prematurity across multiple organ systems, such as necrotizing enterocolitis, patent ductus arteriosus, bronchopulmonary dysplasia, severe retinopathy of prematurity, and preterm brain injury [53]. With respect to the development of the infant brain, the third trimester sees remarkable increases in size and complexity [54,55]. The neuroanatomical effects of preterm birth can be seen into childhood, with children born at younger GA displaying decreased brain volume, surface area, and cortical thickness compared to full-term counterparts [56]. We hypothesize the magnitude of developmental disruption in EP infants, and some of the related comorbidities that contribute to brain injury in this population, may be too significant for MgSO_4_ to compensate for, thus potentially eliminating a protective effect against CP observed in older preterm infants.

Recent research has suggested the presence of a sex-dependent effect of MgSO_4_ on neurodevelopmental outcomes in EP neonates, resulting in worsened outcomes in males [15]. The presence of sexually dimorphic outcomes is well-documented in neonatology [57]. Though very premature males tend to have higher birth weights, they also have higher rates of death, oxygen dependency, pulmonary hemorrhage, postnatal steroid use, and major intracranial abnormalities [58]. Further, males born EP often have lower neurodevelopmental scores in early childhood compared to females, which is hypothesized to be related to differences in the effects of sex hormones and inflammation and cell death pathways [59]. For example, in rat models of neonatal brain injury, 17β-estradiol and progesterone administration were associated with improved cellular and behavioral outcomes, respectively [60,61]. In response to the proinflammatory agent lipopolysaccharide, umbilical cord blood from male pups produced greater amounts of interleukin-6 and interleukin-1β compared to female samples [62]. When considering the effects of Ca^2+^-mediated excitotoxicity on preterm brain injury, mitochondria isolated from male rat neurons have a greater calcium uptake capacity than females, which is consistent with additional findings that males produce more free radical species than females after insult [59]. Consequently, males have higher incidence of CP than females but importantly, there is no significant sex difference in the effectiveness of common treatments for CP such as botulin toxin injection in the lower extremities, use of orthoses in the upper extremities, or single-event multilevel surgery for spastic diplegic CP [63]. Though it is vital to understand and investigate the implications of sex differences, the presence of innate biological differences does not necessitate that the efficacy of a treatment will be modulated by sex, and it is just as important to discuss the absence of a sex-dependent association. In an evaluation of 216 studies investigating sex-effects of treatments for a range of conditions, 99 reported no difference and the vast majority of the 97 articles purporting sex-differences lacked statistical support [64].

As a secondary analysis of a clinical trial in EP infants, our study has some limitations. It is possible that there is a sex effect of MgSO_4_ in less premature infants or emerges at a later age, but we are unable to assess that in this dataset. We are also unable to determine a causal relationship between MgSO_4_ and neurodevelopmental outcomes. While we attempted to minimize the risk of confounding by indication, we cannot eliminate the possibility that an undetected effect remains. Though we utilized well-established measures of neurodevelopmental outcomes, it would be advisable for future studies to assess such outcomes at school age with clinical evaluation for CP. Furthermore, though GMFCS levels are a well-established prognosticator for CP, they do not necessarily equate to a CP diagnosis which may also be contributing to this disparity in results. Finally, dose and timing of MgSO_4_ were not documented and could not be included in our models, which may also account for differences relative to other studies.

This study utilizes one of the largest contemporary cohorts of preterm infants from a multicenter clinical trial and has accounted for 17 maternal and birth covariates that may impact the administration of MgSO_4_, long-term neurodevelopmental outcomes, and CP predictors from standardized assessments. Our use of unadjusted, weighted, and 2:1 matched statistical analysis bolsters the consistent results across all groups. Our results indicate that MgSO_4_ does not have any association with cognitive, motor, language, or CP predicted outcomes for either sex. As survival rates for less mature newborns increase with improved neonatal care, it is vital that our understanding of neuroprotectants and neurocritical care develops alongside this growing and vulnerable population. With an average gestational age of 26 weeks, our study provides a valuable observational foundation for further investigation of MgSO_4_ in the EP population.

## 5. Conclusions

To our knowledge, this study is the first to use a large, multi-center dataset to investigate the sex-dependent associations of MgSO_4_ on outcomes in EP infants while accounting for maternal demographic and clinical covariates contributing to propensity to receive MgSO_4_. Contrary to what has been previously described, we did not observe a sex-dependent association of MgSO_4_ in an EP population of neonates, or any relationship between MgSO_4_ and neurodevelopmental outcomes overall. Though MgSO_4_ appeared to be safe, appropriate clinical trials of MgSO_4_-exposed EP infants are still required to better understand any effects on neurodevelopmental outcomes.

## Figures and Tables

**Table 1:** Infant and maternal characteristics of the study population stratified by (**A**) unadjusted population and (**B**) 2:1 (MgSO_4_ exposed/unexposed) matching. Continuous variables shown as mean (SD) and categorical variables shown as *n* (%). Significant *p*-values (<0.05) shown in bold. To account for correlation within multiple births, *p*-values were calculated using GEE with robust standard errors and Wald tests for both categorical and continuous variables.

Unadjusted (A)	MgSO_4_ Exposure				Matched (B)	MgSO_4_ Exposure			
	No	Yes	All	*p*-Value		No	Yes	All	*p*-Value
N	104	562	666		N	104	208	312	
Maternal age	28.4 (6.6)	29.3 (6)	29.2 (6.1)	0.22	Maternal age	28.4 (6.6)	28.1 (6)	28.2 (6.2)	0.74
Gestational age	25.8 (1.1)	26 (1.2)	26 (1.2)	0.08	Gestational age	25.8 (1.1)	25.8 (1.2)	25.8 (1.2)	0.78
Sex					Sex				
Female	55 (53)	273 (49)	328 (49)	0.46	Female	55 (53)	96 (46)	151 (48)	0.30
Male	49 (47)	289 (51)	338 (51)		Male	49 (47)	112 (54)	161 (52)	
Maternal ethnicity					Maternal ethnicity				
Hispanic	25 (24)	125 (22)	150 (23)	0.52	Hispanic	25 (24)	52 (25)	77 (25)	0.84
Not Hispanic	77 (74)	433 (77)	510 (77)		Not Hispanic	77 (74)	154 (74)	231 (74)	
Unknown	2 (2)	4 (1)	6 (1)		Unknown	2 (2)	2 (1)	4 (1)	
Maternal race					Maternal race				
White	64 (62)	389 (69)	453 (68)	0.54	White	64 (62)	129 (62)	193 (62)	0.98
Black	27 (26)	116 (21)	143 (21)		Black	27 (26)	51 (25)	78 (25)	
Other	9 (9)	36 (6)	45 (7)		Other	9 (9)	18 (9)	27 (9)	
Unknown	4 (4)	21 (4)	25 (4)		Unknown	4 (4)	10 (5)	14 (4)	
Maternal education					Maternal education				
HS or less	31 (30)	181 (32)	212 (32)	0.25	High school or less	31 (30)	63 (30)	94 (30)	0.65
Some college	29 (28)	177 (31)	206 (31)		Some college	29 (28)	71 (34)	100 (32)	
BS or greater	27 (26)	153 (27)	180 (27)		BS or greater	27 (26)	41 (20)	68 (22)	
Unknown	17 (16)	51 (9)	68 (10)		Unknown	17 (16)	33 (16)	50 (16)	
Maternal obesity	5 (5)	67 (12)	72 (11)	**0.04**	Maternal obesity	5 (5)	20 (10)	25 (8)	0.16
Gestational diabetes	4 (4)	34 (6)	38 (6)	0.39	Gestational diabetes	4 (4)	14 (7)	18 (6)	0.34
Maternal hypertension	5 (5)	131 (23)	136 (20)	**<0.001**	Maternal hypertension	5 (5)	12 (6)	17 (5)	0.73
Prenatal care	94 (90)	548 (98)	642 (96)	**0.002**	Prenatal care	94 (90)	197 (95)	291 (93)	0.18
Multiple gestation	30 (29)	140 (25)	170 (26)	0.50	Multiple gestation	30 (29)	55 (26)	85 (27)	0.72
Preterm labor	68 (65)	357 (64)	425 (64)	0.74	Preterm labor	68 (65)	136 (65)	204 (65)	1.00
PROM	26 (25)	155 (28)	181 (27)	0.63	PROM	26 (25)	65 (31)	91 (29)	0.31
Prenatal antibiotics	21 (20)	225 (40)	246 (37)	**0.001**	Prenatal antibiotics	21 (20)	61 (29)	82 (26)	0.12
Prenatal steroids	44 (42)	434 (77)	478 (72)	**<0.001**	Prenatal steroids	44 (42)	111 (53)	155 (50)	0.10
C-section	66 (63)	384 (68)	450 (68)	0.37	C-section	66 (63)	134 (64)	200 (64)	0.88
Small for Gestational Age	4 (4)	83 (15)	87 (13)	**0.01**	Small for Gestational Age	4 (4)	9 (4)	13 (4)	0.83

**Table 2:** Cognitive, motor, and language BSID-III scores in the unadjusted study population and after statistical adjustment by weighting and 2:1 (MgSO_4_ exposed/unexposed) matching. Estimated mean effects and 95% confidence intervals were estimated with GEE, clustering on mother using an independent correlation structure with robust standard errors. Data represented as mean (95% CI).

	Unadjusted (*n* = 666)	*p*-Value	Weighted (*n* = 666)	*p*-Value	Matched (*n* = 312)	*p*-Value
BSID—Cognitive						
Female	−1.67 (−6.9, 3.57)	0.53	0.57 (−8.92, 10.06)	0.91	−0.91 (−7.22, 5.41)	0.78
Male	−0.83 (−6.52, 4.86)	0.78	−1.72 (−7.63, 4.19)	0.57	−2.53 (−8.59, 3.54)	0.41
Interaction	0.84 (−6.84, 8.53)	0.83	−2.29 (−13.44, 8.87)	0.69	−1.62 (−10.1, 6.86)	0.71
BSID—Motor						
Female	−0.18 (−4.79, 4.42)	0.94	3.51 (−3.62, 10.64)	0.33	1.73 (−3.66, 7.13)	0.53
Male	0.95 (−4.79, 6.69)	0.75	−0.05 (−6.16, 6.06)	0.99	−0.38 (−6.48, 5.72)	0.90
Interaction	1.13 (−6.17, 8.43)	0.76	−3.56 (−12.91, 5.79)	0.46	−2.12 (−10.08, 5.84)	0.60
BSID—Language						
Female	−0.15 (−4.37, 4.07)	0.95	0.4 (−4.74, 5.53)	0.88	−0.36 (−5.71, 5)	0.90
Male	1.38 (−4.86, 7.63)	0.66	0.34 (−7.08, 7.77)	0.93	0.15 (−6.53, 6.84)	0.96
Interaction	1.53 (−5.94, 8.99)	0.69	−0.05 (−9.03, 8.92)	0.99	0.51 (−7.72, 8.74)	0.90

**Table 3:** GMFCS level of the study population stratified by sex and by adjustment method, (**A**) unadjusted population and (**B**) 2:1 (MgSO_4_ exposed/unexposed) matching. Level five GMFCS corresponds to the most severe motor limitations. Data represented as *n* (%). *p*-values were calculated using Fisher’s exact tests, and do not account for correlation among multiples within a birth.

Unmatched (A)	No MgSO_4_	MgSO_4_	GMFCS Level	*p*-Value	Matched (B)	No MgSO_4_	MgSO_4_	GMFCS Level	*p*-Value
Female	*n* = 54	*n* = 273			Female	*n* = 54	*n* = 96		
	47 (87)	240 (88)	0	0.65		47 (87)	84 (88)	0	0.54
	3 (6)	14 (5)	0.5			3 (6)	4 (4)	0.5	
	2 (4)	14 (5)	1			2 (4)	7 (7)	1	
	2 (4)	3 (1)	2			2 (4)	1 (1)	2	
	0 (0)	0 (0)	3			0 (0)	0 (0)	3	
	0 (0)	2 (0.7)	4			0 (0)	0 (0)	4	
	0 (0)	0 (0)	5			0 (0)	0 (0)	5	
Male	*n* = 49	*n* = 287			Male	*n* = 49	*n* = 111		
	41 (84)	239 (83)	0	0.74		41 (84)	89 (80)	0	0.74
	2 (4)	18 (6)	0.5			2 (4)	9 (8)	0.5	
	5 (10)	13 (5)	1			5 (10)	6 (5)	1	
	1 (2)	8 (3)	2			1 (2)	4 (4)	2	
	0 (0)	4 (1)	3			0 (0)	2 (2)	3	
	0 (0)	3 (1)	4			0 (0)	1 (1)	4	
	0 (0)	2 (1)	5			0 (0)	0 (0)	5	

## Data Availability

The original data presented in the study are openly available in NINDS Data Archive at: https://www.ninds.nih.gov/current-research/research-funded-ninds/clinical-research/archived-clinical-research-datasets (accessed on 29 October 2025).

## Abbreviations

The following abbreviations are used in this manuscript:

BSID	Bayley Scales for Infant Development 3rd Edition
CI	Confidence interval
CP	Cerebral palsy
EP	Extremely preterm
Epo	Erythropoietin
GA	Gestational age
GEE	Generalized estimating equations
GMFCS	Gross Motor Function Classification System
HI	Hypoxic–ischemic
IL-6	Interleukin-6
MgSO_4_	Magnesium sulfate
NICU	Neonatal intensive care unit
NMDA	*N*-methyl-D-aspartate
OR	Odds ratio
PENUT	Preterm Erythropoietin Neuroprotection Trial
PROM	Premature rupture of membranes
RR	Risk ratio
SGA	Small for gestational age
STROBE	Strengthening the Reporting of Observational Studies in Epidemiology
TNF	Tumor necrosis factor
